# Structures and characteristics of carbohydrates in diets fed to pigs: a review

**DOI:** 10.1186/s40104-019-0345-6

**Published:** 2019-04-17

**Authors:** Diego M. D. L. Navarro, Jerubella J. Abelilla, Hans H. Stein

**Affiliations:** 1 Department of Animal Sciences, University of Illinois, Urbana, 61801 USA; 2 Division of Nutritional Sciences, University of Illinois, Urbana, 61801 USA

**Keywords:** Carbohydrates, Carbohydrate composition, Chemical structure, Feed ingredients, Fiber, Pigs

## Abstract

The current paper reviews the content and variation of fiber fractions in feed ingredients commonly used in swine diets. Carbohydrates serve as the main source of energy in diets fed to pigs. Carbohydrates may be classified according to their degree of polymerization: monosaccharides, disaccharides, oligosaccharides, and polysaccharides. Digestible carbohydrates include sugars, digestible starch, and glycogen that may be digested by enzymes secreted in the gastrointestinal tract of the pig. Non-digestible carbohydrates, also known as fiber, may be fermented by microbial populations along the gastrointestinal tract to synthesize short-chain fatty acids that may be absorbed and metabolized by the pig. These non-digestible carbohydrates include two disaccharides, oligosaccharides, resistant starch, and non-starch polysaccharides. The concentration and structure of non-digestible carbohydrates in diets fed to pigs depend on the type of feed ingredients that are included in the mixed diet. Cellulose, arabinoxylans, and mixed linked β-(1,3) (1,4)-*d*-glucans are the main cell wall polysaccharides in cereal grains, but vary in proportion and structure depending on the grain and tissue within the grain. Cell walls of oilseeds, oilseed meals, and pulse crops contain cellulose, pectic polysaccharides, lignin, and xyloglucans. Pulse crops and legumes also contain significant quantities of galacto-oligosaccharides including raffinose, stachyose, and verbascose. Overall, understanding the structure, characteristics and measurable chemical properties of fiber in feed ingredients may result in more accurate diet formulations, resulting in an improvement in the utilization of energy from less expensive high-fiber ingredients and a reduction in reliance on energy from more costly cereal grains.

## Introduction

Carbohydrates, which are made up of carbon, hydrogen, and oxygen, are organic compounds that serve as a source of energy for animals and humans [1]. The main monosaccharide is glucose, which is utilized as an energy source by animals. Glucose can be derived from starch and sugars in the diet, from glycogen that is stored in the body, or synthesized from the carbon skeleton of amino acids, lactate, glycerol, or propionate via gluconeogenesis [2]. The brain preferentially uses glucose as its main source of energy, and glucose is the required energy source for red blood cells and other cells with few or no mitochondria [3].

The fate of ingested carbohydrates in an animal is determined by the monomeric composition of the carbohydrate, the types of linkages among monomers, and the degree of polymerization (DP) [1]. Digestible carbohydrates include monosaccharides, disaccharides, starch, and glycogen. Only monosaccharides can be absorbed from the small intestine, but glycosidic linkages in disaccharides, starch, and glycogen may be hydrolyzed by endogenous enzymes in the small intestine, resulting in release of their constituent monosaccharides. However, these enzymes show high specificity to their target sugar units, which consequently results in only a limited number of carbohydrates in the feed that can be digested by the animal [2]. Non-digestible carbohydrates that reach the large intestine may be digested by microbial enzymes because intestinal microorganisms secrete glycoside hydrolases and polysaccharide lyases that humans and pigs do not express [4].

Non-digestible carbohydrates include oligosaccharides, resistant starch, and non-starch polysaccharides and are collectively known as fiber [1]. The large differences in the physical properties of carbohydrates make it difficult to analyze fiber and non-digestible carbohydrates [5]. Dietary fiber may be divided according to solubility. Soluble dietary fiber (SDF) may be partially or completely fermented by the microbiota in the large intestine [2], producing short-chain fatty acids (SCFA), which include acetate, propionate, and butyrate [6]. Insoluble dietary fiber (IDF) may also be fermented, but to a lesser extent than SDF [7]. Fermentation of dietary fiber is a major source of energy in ruminants and hindgut fermenters, but only to a lesser extent in pigs and poultry [8]. The relationship between the host and the gut microbiota is symbiotic. As microorganisms ferment non-digestible carbohydrates, endogenous mucosal secretions, and exfoliated epithelial cells to utilize the carbon and N to sustain themselves, SCFA and lactate are produced and absorbed by the animal [4]. The preferred energy source of intestinal microbiota is carbohydrates, but microbes also ferment protein in the absence of carbohydrates, producing branched-chain fatty acids and nitrogenous metabolites such as amines, ammonia, skatole, and indoles [9, 10].

The objective of this contribution is to review the structure and chemical composition of digestible carbohydrates and fiber components in common feed ingredients used in swine diets. The chemical composition of monosaccharides and the monosaccharide composition of dietary fiber in cereal grains, cereal grain co-products, oilseeds and oilseed meals, and in pulse crops are highlighted. It is outside the scope of this review to discuss physical characteristics of fiber or effects of fiber on nutrient digestibility, fermentability, intestinal health, and intestinal microbial activity although it is recognized that these topics also contribute to the overall nutritional value of dietary fiber.

## Definition of carbohydrates

Classification according to molecular size or DP groups carbohydrates into monosaccharides, disaccharides, oligosaccharides, and polysaccharides [1]. Monosaccharides are chiral, polyhydroxylated aldoses or ketoses that cannot be hydrolyzed into smaller carbohydrate units [11]. They can be classified according to the number of carbon atoms in their structure, which range from three to nine carbon atoms (i.e., triose, tetrose, pentose, hexose, heptose, octose, and nonose), by the type of carbonyl group they contain (i.e., aldose or ketose), and by their stereochemistry (i.e., *d* or *ʟ*), and they have the general chemical formula (CH_2_O)_*n*_ [12]. Aldoses are referred to as reducing sugars because of their reducing effect on certain ions or compounds, oxidizing their aldehyde group to a carbonyl group [11]. The simplest aldose sugar with a chiral atom is glyceraldehyde, with its second C molecule attached to four different groups, giving the ability for this C to have two spatial configurations, and glyceraldehyde therefore exist in both the *d*- and the *ʟ*- forms [2]. Chiral carbon atoms have each of their four tetrahedral bonds connected to a different group [13]. The chirality of sugars and AA are commonly designated by the *d*/*ʟ* system and is named in relation to the structure of glyceraldehyde [2].

### Monosaccharides

**Fig. 1:**
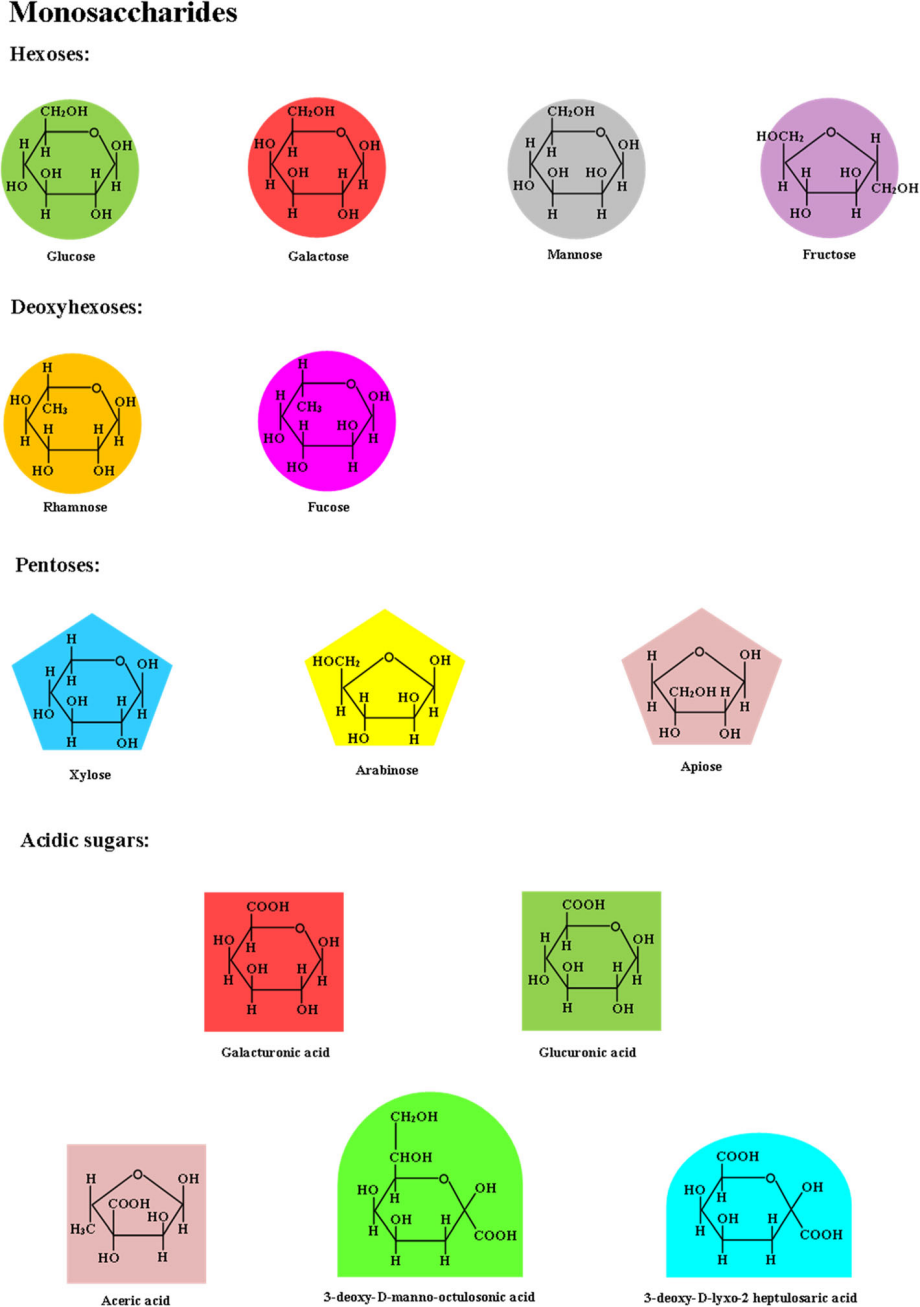
Chemical structure of monosaccharides that are commonly associated with digestible carbohydrates and fiber. Adapted from Albersheim et al. [40]

The most common monosaccharides are the 6-C aldohexoses, which include the aldohexose *d*-glucose, and are usually present in their ring structures called a pyranose ring rather than in open-chain structures (Fig. 1) [11]. In oligo- and polysaccharides, aldopentoses can occur as a 5-C ring structure known as a furanose ring [11]. *d*-Glucose, considering all of its combined forms, is the most abundant monosaccharide that naturally occurs in nature [13]. The most abundant ketose is d-arabino-hexulose, known more commonly by its trivial name, *d*-fructose [2]. The three trioses include ketose dihydroxyacetone and both enantiomeric forms of glyceraldehyde [14]. Erythrose and threose are examples of tetroses, and pentoses include ribose, arabinose, xylose, and apiose [2].

Sugars, such as glucose, galactose, mannose, and fructose, which have different structures, but have the same chemical formula, C_6_H_12_O_6_, are called isomers [3]. Sugars that differ in configuration around only one carbon atom are called epimers, such as *d*-glucose and *d*-mannose, which vary in their structures around C-2 [2]. A pair of enantiomers is a special type of isomerism where the two members of the pair are mirror images of each other and are designated as being in the *d*- or *ʟ*- structure (i.e., *d*-glucose or *ʟ*-glucose), depending on the position of the –OH group linked to the asymmetric carbon farthest from the carbonyl group [3].

Other types of monosaccharides include alditols, or polyols, which are aldoses or ketoses that had their carbonyl groups reduced to an alcohol [13]. An example of a naturally occurring alditol in plants and other organisms is *d*-glucitol, known commonly as sorbitol, which is the product of the reduction of *d*-glucose [13]. Absorption and metabolism of polyols vary among types, but most are fermented in the large intestine [15].

Deoxy sugars are missing one or more hydroxyl groups attached to their carbon atoms, such as 6-deoxy-*ʟ*-mannose (*ʟ*-rhamnose), which is commonly associated with pectin, 2-deoxy- *d*-ribose, the sugar component of DNA, and 6-deoxy-*ʟ*-galactose (*ʟ*-fucose), a component of glycoproteins and glycolipids in cell walls and mammalian cells [13, 14, 16].

Uronic acids are sugar acids in which the terminal –CH_2_OH group undergoes oxidation to yield a carboxylic acid [14]. Uronic acids that contribute to dietary fiber include constituents of non-digestible polysaccharides of plants and algae, such as *d*-glucuronic acid, *d*-galacturonic acid, *d*-mannuronic acid, and *ʟ*-guluronic acids [2]. Sugar from the activated form of glucuronic acid is used in the synthesis of glycosaminoglycans in mammals, and *ʟ*-iduronic acid is synthesized from *d*-glucuronic acid after it has been incorporated into the carbohydrate chain [3].

### Disaccharides

**Fig. 2:**
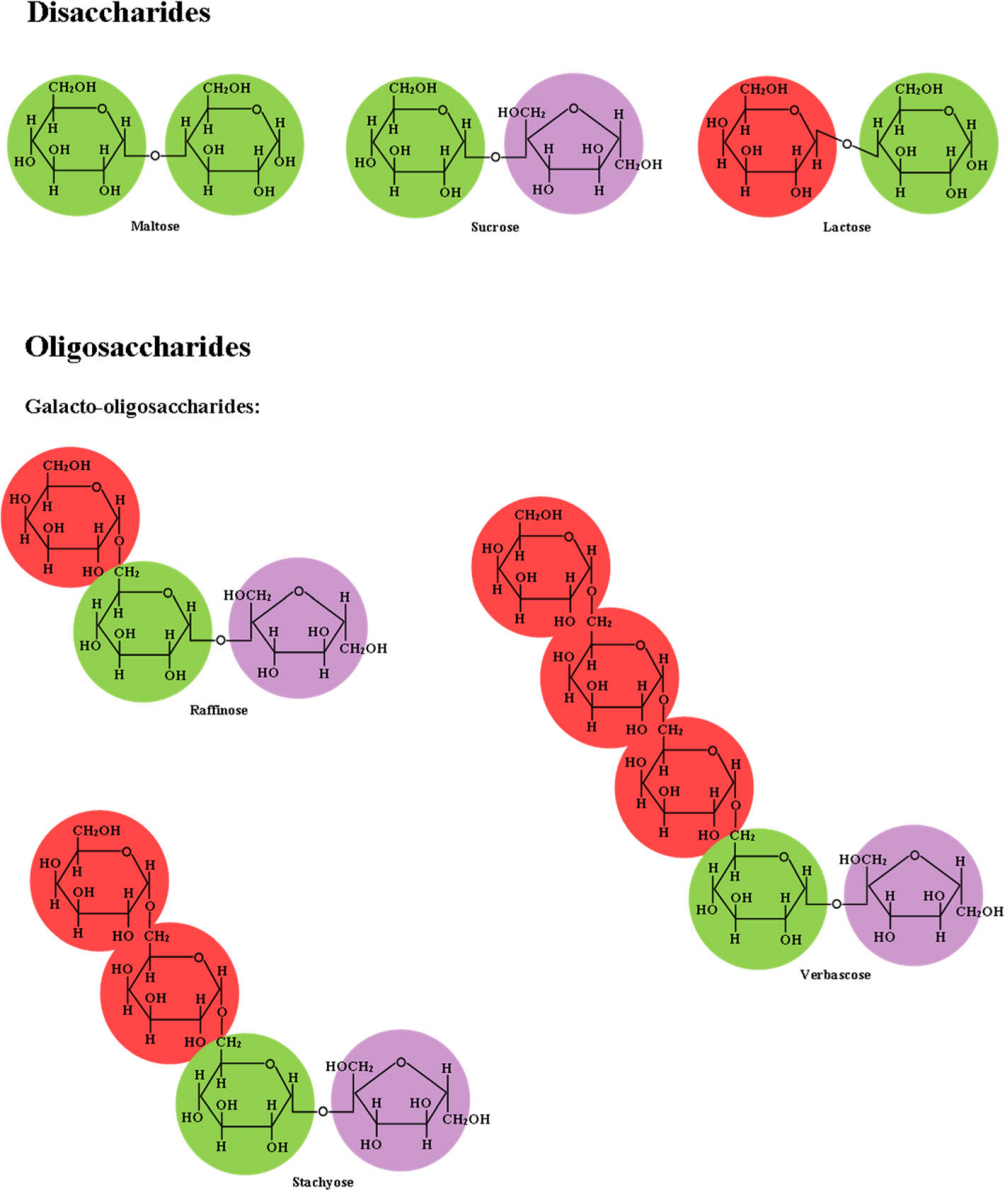
Chemical structure of di- and oligosaccharides. Adapted from Bach Knudsen et al. [1]

Two monosaccharide units joined by an acetal or ketal linkage is referred to as a disaccharide [14]. A glycosidic bond joins 2 monosaccharide units and it can either be an α-glycosidic bond if the anomeric hydroxyl group of the sugar is in the α configuration or a β-glycosidic bond if it is in the β configuration [3]. A glycosidic bond is named according to the position of the carbon atom being linked, for example, an α-glycosidic bond connecting C-1 of a glucose molecule and C-4 of another glucose molecule in maltose is called an α-(1,4) glycosidic bond (Fig. 2) [17]. The three most common disaccharides are maltose, lactose, and sucrose [11]. Maltose is a reducing sugar that is a product of the hydrolysis of starch by the enzyme α-amylase [13]. Lactose is a reducing sugar that consists of a *d*-glucosyl unit and an α-*d*-galactopyranosyl unit linked by a β-(1,4) glycosidic bond and is present in milk and milk products such as skim milk and whey [17]. Sucrose is made up of a glucose and a fructose linked by an α-(1,2) glycosidic bond [17]. Contrary to the general head-to-tail linkage (anomeric carbon atom to carbon atom containing a hydroxyl group) in the structure of oligo- and polysaccharides, in sucrose the glycosidic bond linking an α-*d*-glucopyranosyl unit and a β-*d*-fructofuranosyl unit is in a head-to-head fashion (anomeric carbon atom to anomeric carbon atom) making it a non-reducing sugar [13]. Sucrose is synthesized through the process of photosynthesis to provide energy and carbon atoms for the synthesis of other compounds in the plant [13].

Maltose, lactose, and sucrose are hydrolyzed into their constituent monosaccharide units by the enzymes maltase, lactase, and sucrase, respectively [17]. The α-glucosidases maltase-glucoamylase and sucrase-isomaltase complexes that are present in the brush border of the small intestine cleave the glycosidic bonds in maltose and sucrose, respectively, with most of the maltase activity coming from the sucrase-isomaltase complex [2, 13, 17]. The monosaccharides that result from the digestion of these disaccharides are readily absorbed in the small intestine [18]. Lactase, a β-galactosidase, also is expressed by young mammals that digest lactose into its constituent monosaccharides that are subsequently absorbed in the small intestine [1, 13].

Other disaccharides that are present in nature include trehalose, cellobiose, and gentiobiose [17]. Trehalose is a nonreducing disaccharide made up of two α-*d*-glucopyranosyl units linked together by an α-(1,1) glycosidic bond [2]. Trehalose is found in small amounts in mushrooms, yeasts, honey, certain seaweeds, and invertebrates such as insects, shrimps, and lobsters [13]. Trehalose is digested by the α-glucosidase enzyme trehalase, which is expressed in the small intestine of humans and most animals [2]. Two glucose molecules are linked together by a β-(1,4) and a β-(1,6) glycosidic bonds to form cellobiose and gentiobiose, respectively, and these disaccharides can be utilized only after microbial fermentation because pigs lack the enzymes capable of digesting these bonds [17]. Cellobiose is a product of cellulose degradation, whereas gentiobiose is believed to play a role in the initiation of ripening of tomato fruits [19].

### Oligosaccharides

Oligosaccharides consist of galacto-oligosaccharides, fructo-oligosaccharides, and mannan-oligosaccharides that cannot be digested by pancreatic or intestinal enzymes, but are soluble in 80% ethanol [15, 20]. Galacto-oligosaccharides, or α-galactosides, that are present in large amounts in legumes, are comprised of raffinose, stachyose, and verbascose, which have a structure consisting of a unit of sucrose linked to one, two, or three units of d-galactose, respectively (Fig. 2) [2]. These oligosaccharides cause flatulence in pigs and humans due to the lack of an enzyme, α-galactosidase, that hydrolyzes the glycosidic bonds linking the monosaccharides that constitute these α-galactosides and are, therefore, utilized by bacteria in the large intestine [12, 21]. In raffinose, *d*-galactose is linked to sucrose by an α-(1,6) bond, whereas two units and three units of *d*-galactose are linked to sucrose, also via α-(1,6) glycosidic bonds, in stachyose and verbascose, respectively [17]. Transgalacto-oligosaccharides are another type of galacto-oligosaccharides that may have prebiotic effects in young pigs and are commercially synthesized from the transglycosylation actions of β-glycosidases on lactose, creating β-(1,6) polymers of galactose linked to a terminal glucose unit via an α-(1,4) glycosidic bond [17, 22]. However, transgalacto-oligosaccharides are not naturally synthesized [17].

Fructo-oligosaccharides, or fructans, are chains of fructose monosaccharides with a terminal glucose unit and are classified as inulins or levans [17, 23]. Inulin is mostly found in dicotyledons, whereas levans are mainly found in monocotyledons [24]. Fructo-oligosaccharides are not hydrolyzed in the small intestine due to the β-linkages between their monomers, but can be fermented to lactic acid and SCFA in the large intestine [2, 20, 25]. Inulin occurs naturally in onions, garlic, asparagus, bananas, Jerusalem artichoke, wheat, and chicory as a storage carbohydrate [13, 15, 20]. Inulin is made up of β-*d*-fructofuranosyl units linked by β-(2,1) glycosidic linkages and have a DP that ranges from 2 to 60 [13, 17]. The polymer is composed of fructose residues present in the furanose ring form and often have a terminal sucrose unit at the reducing end [2, 13]. Levans are fructans that have an average length of 10 to 12 fructose units linked by β-(2,6) linkages, but can have a DP of more than 100,000 fructose units and are found in bacterial fructans and in many monocotyledons [24, 26]. Levans are derived from the transglycosylation reactions catalyzed by the enzyme levansucrase that is secreted by certain bacteria and fungi that preferentially use the *d*-glycosyl unit of sucrose, thereby converting sucrose to levans with β-(2,1) linked side-chains [13, 17]. Polysaccharides containing a significant number of β-(2,1) linkages also can be referred to as “levan” [14]. A third type of fructans, called graminan-type fructans, contain a combination of both β-(2,1) and β-(2,6) linkages and are present in wheat and barley [27].

Mannan-oligosaccharides are composed of polymers of mannose that are derived from yeast cell walls, and are located on the outer surface of yeast cell walls attached to β-glucans of the inner matrix via β-(1,6) and β-(1,3) glycosidic linkages [17]. Mannan-oligosaccharides and fructo-oligosaccharides may behave as prebiotics due to their beneficial health effects on the host by stimulating the growth or activity of certain bacteria in the large intestine [28]. It has been suggested that mannan-oligosaccharides regulate the response to immunological challenges by pigs and may prevent overstimulation of the host animal’s immune system following an infection [29].

### Polysaccharides

Polysaccharides are high-molecular-weight carbohydrates that are polymers of monosaccharides [13]. Polysaccharides are made up of sugar polymers that vary in size and may either be linear or branched [2]. The DP varies with the type of polysaccharide and may range from 7,000 to 15,000 in cellulose and up to more than 90,000 in amylopectin [13]. Polysaccharides can be classified as homopolysaccharides if they contain only one type of sugar residue (e.g., starch, glycogen, and cellulose) or as heteropolysaccharides if they contain two or more different kinds of sugar residues in their structure (e.g., arabinoxylans, glucomannans, and hyaluronic acid; 2). Polysaccharides are present in large quantities in pig diets, and are divided into starch and glycogen and non-starch polysaccharides (NSP) [17, 30].

**Fig. 3:**
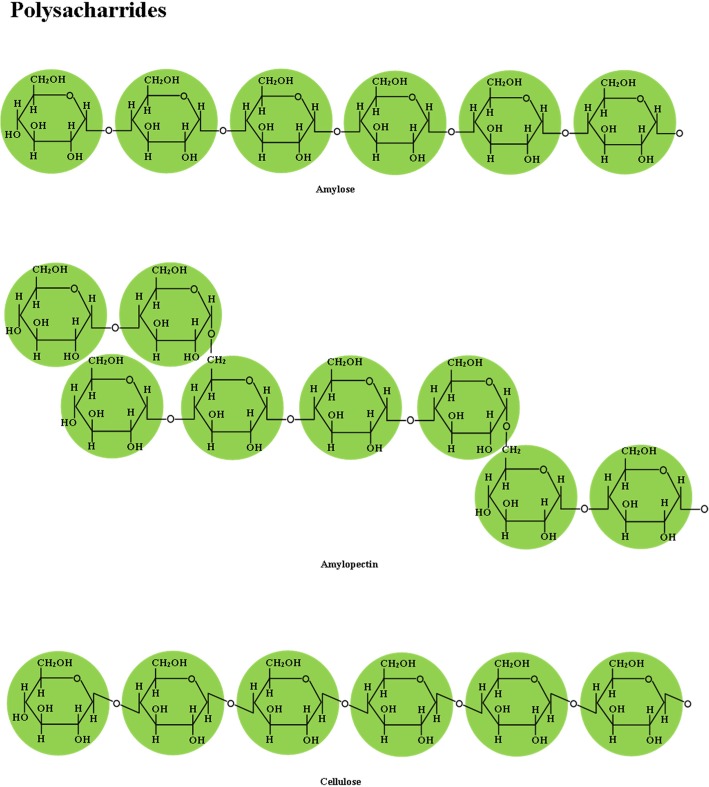
Chemical structure of amylose, amylopectin, and cellulose. Adapted from Bach Knudsen et al. [1]

Starch can be linear or branched and is the storage form of carbohydrates in plants, whereas glycogen is highly branched and is present only in animal tissue, primarily in the muscle and liver [2, 31]. Starch is one of the most abundant carbohydrates in nature [2]. It is synthesized to store energy for plant growth and is stored in seeds, tubers, roots, stems, leaves, and some fruits [32]. Starch is a polymer of *d*-glucose that is comprised of two types of molecules, amylose and amylopectin (Fig. 3) [12]. Amylose is a short linear polymer of glucose with an average DP of 1,000 glucose units linked via α-(1,4) bonds. Amylopectin contains larger chains of glucose with DP of 10,000 to 100,000 with branch points at the α-(1,6) linkages for every 20 to 25 glucose units [15, 30]. The total number of α-(1,6) linkages are only about four to five % of the total glycosidic bonds in amylopectin [33]. Native starch contains both forms as semi-crystalline granules of varying proportions of amylose and amylopectin, depending on the plant source [30, 31]. Starch granules have varying structural and chemical compositions depending on the plant species and the part of the plant where it is located [18]. The size of the starch granules influences the surface-to-volume ratio, and the smaller the granule, the larger the surface-to-volume ratio resulting in more surface area for enzyme hydrolysis in the digestive tract [30]. Digestion of starch begins in the mouth where salivary α-amylase is secreted, which acts only on the α-(1,4) linked linear chains of amylose and amylopectin, until this enzyme is deactivated by the low pH in the stomach [31]. Large quantities of pancreatic α-amylase specific only to α-(1,4) linkages are secreted into the duodenal lumen, producing maltose and maltotriose as the products of luminal amylose and amylopectin digestion, along with the branched oligosaccharide α-dextrin resulting from the partial hydrolysis of amylopectin due to the inability of α-amylase to cleave α-(1,6) linkages [18]. Starch digestion is completed by oligosaccharidases (i.e., α-glucosidases) expressed by glands in the small intestine. These α-glucosidases include sucrose-isomaltase and maltase-glucoamylase complexes [34]. Both complexes have differences in their degree of specificity for the products of α-amylase digestion and cleave the α-(1,4) and α-(1,6) bonds in α-dextrins in a complementary manner, producing free glucose that is transported into the enterocytes [18].

Starch can be divided into three types: Type A starch has an open structure and is present in cereals; Type B starch is present in tubers and appears to be more compact; and Type C starch is a combination of types A and B starch and is present in legumes [30]. Starch granules in raw potatoes and green bananas that have high amylose content result in more tightly packed granules that are more insoluble and resistant to digestion compared with amylopectin-containing granules that are more branched and less tightly packed [2]. In corn, wheat, and potato, starch may contain approximately 20% amylose and 80% amylopectin [31]. However, waxy corn may have starch containing nearly 100% amylopectin, whereas high amylose corn may contain up to 75% amylose [35]. Therefore, starch may not always be digested by α-amylase unless the cereal grains are altered by physical processing (e.g., grinding or roller milling) and heating (e.g., pelleting, expansion, or extrusion) [30].

A proportion of the starch is not digested by α-amylase or the enzymes of the brush border and may undergo microbial fermentation in the large intestine; this is referred to as resistant starch (RS) [13, 31]. Starch may resists digestion because it is physically inaccessible due to enclosure within whole plant cells or matrices (i.e., RS-1). Native or uncooked starch (RS-2) also resists digestion because of the ungelatinized crystalline structure of the granule, and retrograded starch (RS-3) resists digestion because it is rapidly cooled after it has been gelatinized via heating. If starch is chemically modified, it may also resist digestion and is referred to as RS-4 [13, 30, 31]. Resistant starch serves as a substrate for colonic fermentation but regardless of the amount entering the hindgut, starch is usually fully fermented in the hindgut [25]. Starch-containing ingredients will naturally contain RS, but the amount and type of starch will influence the proportion of total starch that is RS [36]. Processing may influence the proportion of starch resistant to digestion and RS values typically range from 0 to 19% in most cereal grains and 10% to 20% in legumes (Table 1) [15, 37]. Cooking or ripening decreases the quantity of RS in raw or immature fruits or vegetables such as green bananas and potatoes [38].

**Table 1:** Carbohydrates and lignin in cereal grains (g/kg DM)^a, b^

Items	Corn	Wheat	Barley	Oats	Rye	Sorghum	Polished rice	Triticale
Total MBG	1	10	41	28	17	1	0.4	7
Total AX	47	73	84	97	95	24	26	85
A:X	0.74	0.62	0.48	0.22	0.66	1.23	–	0.71
Starch	680	647	587	468	613	585	837	727
Resistant starch	10	4	55	54	12	162	3	–
Cellulose	20	18	43	82	14	14	3	21
Lignin	11	18	35	66	21	24	8	20
Pectin	11	3	3	–	–	19	3	–
Dietary fiber^c^								
Total dietary fiber	127	131	81	73	13	103	13	–
Insoluble dietary fiber	119	126	–	–	–	–	–	–
Soluble dietary fiber	8	5	–	–	–	–	–	–

^a^All values except values for dietary fiber were adapted from McCleary and Glennie-Holmes [63], Bach Knudsen [48], Bailoni et al. [64], Izydorczyk and Biliaderis [50], Bach Knudsen [30], NRC [17], Bach Knudsen [42], and Cervantes-Pahm et al. [37]

^b^*MBG* mixed linked β-glucan, *AX* arabinoxylan, *A:X* arabinose to xylose ratio

^c^Values for dietary fiber were adapted from Cervantes-Pahm et al. [37], Jaworski et al. [65], Navarro et al. [66], Navarro et al. [67]

Glycogen, an α-(1,4)-*d*-glucan with α-(1,6) linked branches, has a higher degree of branching compared with amylopectin and is present in animal tissues, mainly in skeletal muscle and the liver [2]. As a consequence, only pigs fed diets containing animal products will consume glycogen. The branch points of glycogen occur after an average of 8 to 10 glycosyl units [3]. A polymer of glycogen may contain up to 100,000 units of glucose [39]. Digestion of glycogen is similar to that of amylopectin, which results in glucose absorption in the small intestine [17]. The extensive branching of glycogen enhances its solubility, which allows glucose to be mobilized more readily [34].

### Nonstarch polysaccharides

Nonstarch polysaccharides are mainly present in primary or secondary plant cell walls and consist of both soluble and insoluble polysaccharides that unlike starch do not contain α-(1,4)-linked glycosyl units [15, 30]. Primary cell walls surrounding growing cells are mainly composed of polysaccharides and some structural proteins, whereas mature cells that have already differentiated are surrounded by secondary cell walls that also contain polysaccharides and proteins, along with lignin and a larger amount of cellulose [40]. The cell wall polysaccharides consist of pentoses (i.e., arabinose and xylose), hexoses (i.e., glucose, galactose, and mannose), 6-deoxyhexoses (i.e., rhamnose and fucose), and uronic acids (i.e., glucuronic and galacturonic acids) [41]. These components can exist in their pyranose and furanose forms and form α- or β- linkages at any of their available hydroxyl groups resulting in a broad range of functional surfaces by adapting numerous 3-dimensional shapes [42]. Phenolic residues of lignin or its hydroxyl side-chains can also bond with glycosidic linkages of NSP [40]. Nonstarch polysaccharides may acquire hydrophobic properties by linking to lignin and suberin, whereas the degree of esterification of uronic acids may influence its ionic properties [30]. Suberin, a hydrophobic complex mixture of hydroxylated fatty acids and fatty esters, is present in vascular tissues that provide an insoluble barrier during normal development and in response to wounding or fungal infections [40]. Nonstarch polysaccharides also may be classified as soluble and insoluble, where the term soluble refers to solubility of the NSP in water or weak alkali solutions [41].

The most common NSPs in cell walls are cellulose and non-cellulosic polysaccharides (NCP) [17]. On average, the cellulose content of primary cell walls is 20% to 30%, whereas secondary cell walls can contain up to 50% cellulose [40]. Primary cell walls are deposited between the middle lamella and the plasma membrane during cell growth, whereas certain specialized cells deposit a thicker inner layer called the secondary cell wall at the onset of differentiation [43]. Cellulose consists of linear β-(1,4)-linked *d*-glucopyranosyl units with a DP that varies from 500 to 14,000. The linear units of cellulose are stabilized by hydrogen bonding between adjacent glucose residues, forming an organized arrangement of cellulose molecules within the microfibrils (Fig. 3) [42, 44]. Crystalline regions are formed when highly organized cellulose microfibrils are aligned parallel to each other to allow for maximal hydrogen bonding, whereas paracrystalline or amorphous sections are formed in regions that are less organized [45]. The 3-dimensional lattice formed of the closely packed linear and unbranched structure of cellulose forms the microfibrils that give the structure of plant cell walls [46]. The less organized amorphous regions of cellulose are hydrolyzed by endoglucanases, producing chain ends that are hydrolyzed by exoglucanases (i.e., cellobiohydrolases) [45]. The resulting disaccharide, cellobiose, is hydrolyzed by β-glucosidase to produce two glucose monomers [44].

Highly branched NCP consist of heteropolymers of pentoses and hexoses, the most common of which is called a xylan, or a chain of β-(1,4) linked d-xylopyranosyl units with side-chains that are commonly composed of *ʟ*-arabinofuranosyl, *d*-galactopyranosyl, *d*-glucuronopyranosyl, and/or 4-O-methyl-*d*-glucuronopyranosyl units [13]. Non-cellulosic polysaccharides may also contain uronic acids that are derived from glucose and galactose, giving the ability to form salts with Ca and Zn [46]. Non-cellulosic polysaccharides often serve as structural polysaccharides in plant tissues and are closely associated with cellulose and lignin [45].

Lignin is not a carbohydrate, but is associated with cell wall polysaccharides [1]. It consists of polymerized phenylpropane units (i.e., coniferyl, p-coumaryl, and sinapyl alcohols) linked by ether and carbon-carbon bonds in an irregular 3-dimensional pattern [42]. A lignified cell wall may consist of a thin primary layer, followed by a thick multilamellar secondary layer that is high in cellulose, and possibly a third layer [47]. Lignin may link to polysaccharides by forming covalent bonds with sugar residues or ferulic acids that are esterified to these polysaccharides [1]. Lignification occurs only after cell division, cell expansion, and cell elongation have ceased and, therefore, constitutes terminal differentiation, which is typically followed by programmed cell death [40]. Lignin prevents biochemical degradation and physical damage to cell walls by cementing and anchoring cellulose microfibrils and other matrix polysaccharides, hence, enforcing the structural integrity of the cell wall [48]. Lignin also serves as a barrier to pathogens and pests [40]. Plant tissues become lignified or woody when the lignin concentration is high [49]. Lignin is more concentrated in the outer husk layer of grains compared with endosperm cell walls as is evident in the elevated concentrations in ingredient byproducts (Table 2).

**Table 2:** Carbohydrates and lignin in cereal grain byproducts (g/kg DM)^a, b^

Items	Bran					Hulls		Middlings		DDGS^c^	
	Corn	Wheat	Rye	Rice	Oat	Barley	Oat	Wheat	Rye	Corn	Sorghum
Total MBG	2	24	45	–	–	16	14	26	37	–	–
Total AX	207	232	292	–	–	235	240	–	–	–	–
A:X	0.61	0.58	0.36	–	–	0.28	0.13	–	–	–	–
Starch	376	220	–	287	–	172	–	575	369	17	28
Resistant starch	–	2	–	–	–	2	–	–	–	–	–
Cellulose	89	72	39	166	–	192	196	19	27	102	85
Lignin	30	75	68	–	–	115	148	11	39	29	99
Pectin	–	4	–	79	–	–	–	2	–	–	–
Dietary fiber^d^											
Total dietary fiber	457	414	–	–	237	–	–	410	–	423	371
Insoluble dietary fiber	406	376	–	–	115	–	–	390	–	411	329
Soluble dietary fiber	52	38	–	–	113	–	–	21	–	12	42

^a^Adapted from Bach Knudsen [48], Bailoni et al. [64], Bach Knudsen [30], NRC [17], Bach Knudsen [42], Cervantes-Pahm et al. [37], Curry et al. [68], and Jaworski and Stein [69]

^b^*MBG* mixed linked β-glucan, *AX* arabinoxylan, *A:X* arabinose to xylose ratio

^c^Distillers dried grains with solubles

^d^Values for dietary fiber were adapted from McCleary et al. [70], Jaworski et al. [65], Jaworski and Stein [69], Navarro et al. [66], Navarro et al. [67]

## Nonstarch polysaccharides in feed ingredients

### Cereal grains and cereal co-products

**Fig. 4:**
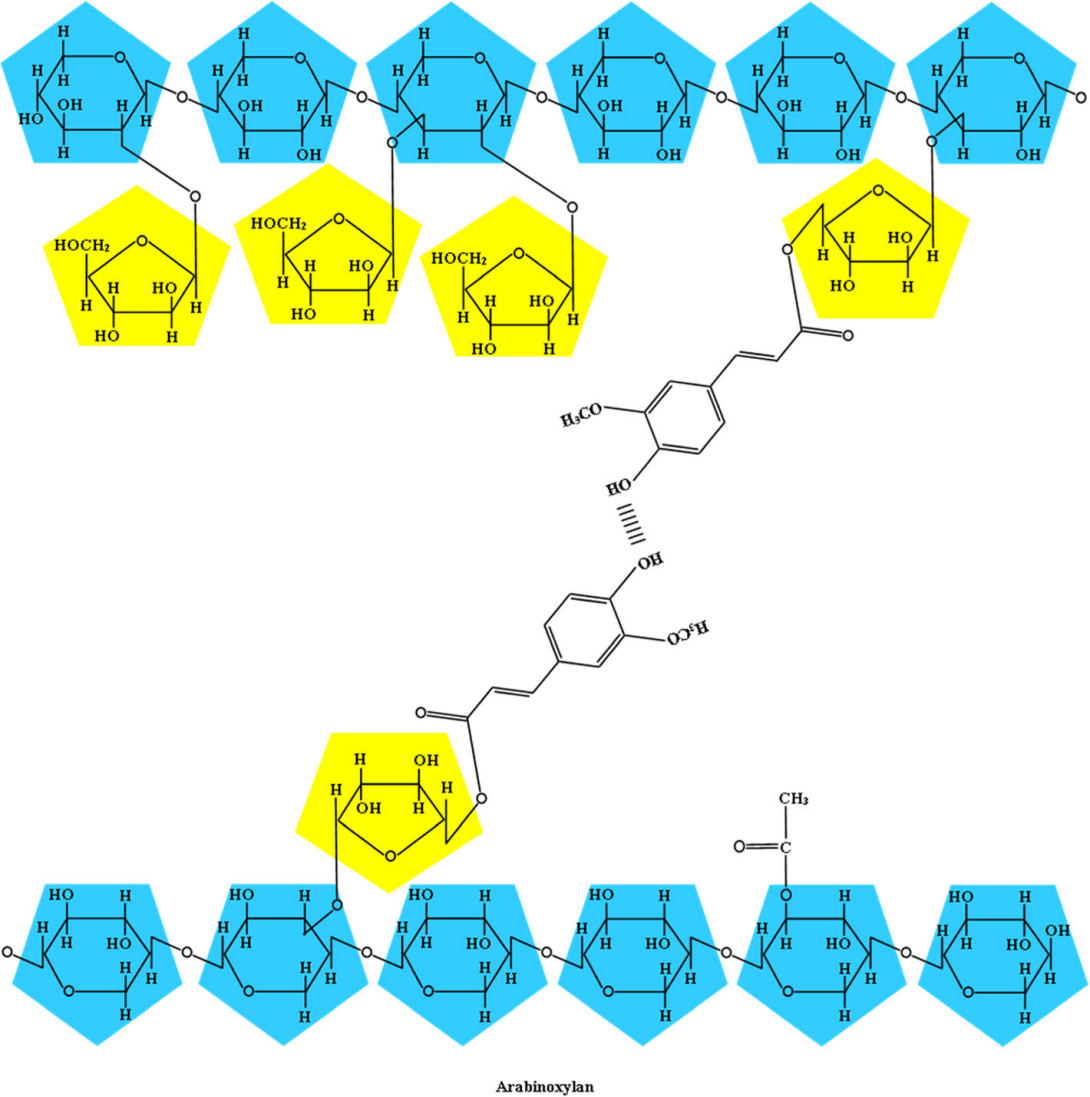
Chemical structure of arabinoxylans linked via a diferulic acid linkage. Adapted from Izydorczyk and Dexter [53] and Bach Knudsen [42]

In cereal grains, the proportion of total cell wall polysaccharides is influenced by several factors including genetics, climate, stage of maturity, the use of nitrogen fertilizers, and post-harvest storage time [45]. Cellulose, mixed linked β-(1,3) (1,4)-*d*-glucans (i.e., β-glucan; MBG), and arabinoxylans (AX) are the main cereal grain cell wall polysaccharides that have varying proportions and structures depending on the species and tissue of the grain (Table 1) [30, 42]. Arabinoxylan has a linear backbone of β-(1,4)-*d*-xylopyranosyl units with varying degrees of α-*ʟ*-arabinofuranosyl residue substitutions and is the main polymer of cell walls in cereals such as corn, wheat, rye, and triticale (Fig. 4) [42]. The α-*ʟ*-arabinofuranosyl residue substitutions can occur at the O-2, O-3, or both O-2 and O-3 of the xylopyranosyl unit, resulting in unsubstituted, monosubstituted, and disubstituted xylose residues in the xylan backbone [14, 50]. This polysaccharide is commonly referred to as a pentosan because it mainly contains pentose sugars [33]. Oats have the greatest concentration of total AX among the cereal grains followed by rye and triticale, whereas sorghum and rice contain the least (Table 1).

Arabinoxylans are primarily located in the cell walls of the endosperm, but may also be present in the outer layers where the structure of AX differs in that glucuronic acid and galactose are also present [42, 51]. These acidic AX are called glucuronoarabinoxylans and are present in the husk and bran of cereal grains [50]. There also may be differences in the structures and characteristics of AX within the grain and among plant species, such as the arabinose to xylose ratio, the sequence and proportions of the various linkages in the structure, and the composition of substituents of the side-chains [52]. The AX in wheat and rye has a larger proportion that is soluble compared with the AX in barley and oats, mainly due to differences in their structural features [42]. Arabinoxylans in the aleurone layer, a specific tissue of cereal endosperm that is structurally similar to the starchy endosperm, may encapsulate available nutrients [42]. The aleurone layer contains ferulic and dihydrodiferulic acids, as well as AX that are more esterified than AX in the starchy endosperm [42]. An ester linkage covalently links ferulic acid to the O at C-5 of the arabinose residue [52]. Ferulic acid can dimerize into dehydrodiferulate esters because of its capability to form both ester and ether linkages, allowing cross-linking between AX chains and between AX and other components of the cell wall [53]. Cereal grain AX are mostly water-insoluble due to alkali-labile cross-linkages between AX and the cell wall; however, AX that are not bound to other cell wall polysaccharides can absorb water and form highly viscous solutions [54]. One-third of the fraction of AX in wheat and rye is soluble in water and this proportion is larger compared with that in barley and oats [42, 45]. The ability to bind water decreases when AX loses arabinose side-chains and, therefore, becomes less soluble [54]. The arabinose to xylose ratio is lower in the insoluble aleurone AX compared with the starchy endosperm of wheat and barley [42]. Of the cereal grains, sorghum has an arabinose to xylose ratio that is greater than 1:1, whereas oats has a ratio that is less than 0.25:1, indicating that sorghum can bind more water and is more soluble compared with oats (Table 1). Furthermore, unsubstituted regions of the backbone of AX may form intermolecular hydrogen bonding between adjacent xylopyranosyl residues, but steric hindrance imposed by arabinose side-chains limit aggregation of AX [52, 54].

**Fig. 5:**
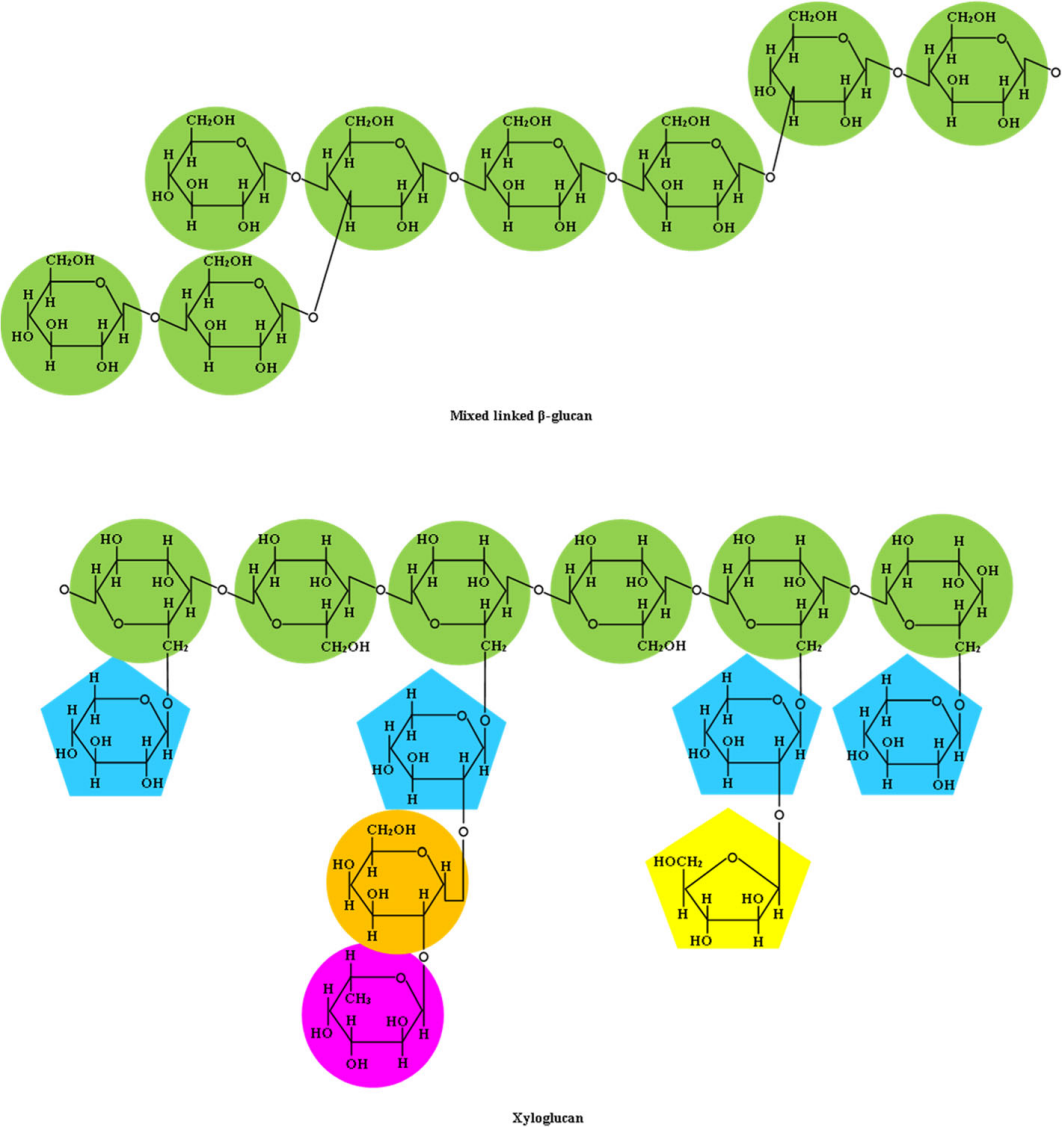
Chemical structure of mixed linked β-glucan and xyloglucan. Adapted from Bach Knudsen et al. [1]

Whereas the main NCP in all cereal grains is AX, concentrations of MBG are 1% or less in corn, wheat, sorghum, triticale, and polished rice. However, rye contains 1.7% MBG, and concentrations of MBG in oats and barley are between 2.8% and 5.0% (Table 1) [45]. Rice, corn, and sorghum have the least concentration of total MBG. Mixed linked β-glucans in cereal grains are soluble linear homopolymers of *d*-glucopyranosyl residues that are linked by two to three consecutive β-(1,4) linkages and separated by a single β-(1,3) linkage (Fig. 5) [42, 45]. Mixed linked β-glucans are soluble in water because of the presence of 2 types of linkages, which prevent the compact folding of the β-glucan chains [25]. There is currently no evidence of MBG containing two or more adjacent β-(1,3) linkages [53]. The general molecular structure of MBG is the same across different genera of cereals, but vary in features such as molecular size, the ratios of β-(1,4) to β-(1,3) linkages, the level of long cellulose-like fragments, and the ratios of trimers to tetramers [42, 55]. Genetic and environmental factors play a role in the differences in the ratio of cellotriosyl to cellotetraosyl units between different varieties within the various cereal grains [42]. Typically, the ratio of β-(1,4) to β-(1,3) bonds is approximately three to two [33]. For example, the structure of MBG in barley consists primarily of cellotriosyl units linked by β-(1,4) bonds and β-(1,3) linked cellotetraosyl units [45]. Dry conditions and warmer temperatures before harvest or during growing time results in high levels of MBG [55]. Barley, oats, and rye contain more MBG in the endosperm, aleurone, and subaleurone cell walls compared with corn and wheat [6, 42, 48]. In barley, the amount of water-soluble MBG is more than four times that of AX, whereas in rye, the concentration of AX is at least three times that of MBG [45]. There is no correlation between total MBG, AX, or NSP and starch content [51].

### Oilseeds and oilseed meals

The cell walls of oilseeds primarily contain cellulose, pectic polysaccharides, lignin, and xyloglucans that serve to protect the seeds [42]. The more complex composition of primary cell walls of protein sources such as soybean cotyledons include rhamnogalacturonans, cellulose, xyloglucans, glycoproteins, arabinans (in rapeseed), and arabinogalactans (in soybeans and rapeseed) that can be present as free arabinogalactans or linked to rhamnogalacturonans [30]. Xyloglucans have a backbone of β-(1,4)-glucosyl units similar to that of cellulose, containing side-chains of xylose, galactose, fucose, and arabinose, with approximately 75% of the β-*d*-glucosyl residues substituted with a single α-*d*-xylosyl residue at the C-6 position (Fig. 5) [40, 56]. Many of the α-*d*-xylosyl residues are substituted at C-2 with glycosyl residues, further extending the side chain [57]. Xyloglucans are strongly associated with cellulose microfibrils in the walls of growing plant cells, forming xyloglucan bridges between the microfibrils [40]. However, variation exists in the structure of xyloglucans among plant species, tissues, cell types and, possibly, even in different parts of the cell wall surrounding individual cells [57].

**Fig. 6:**
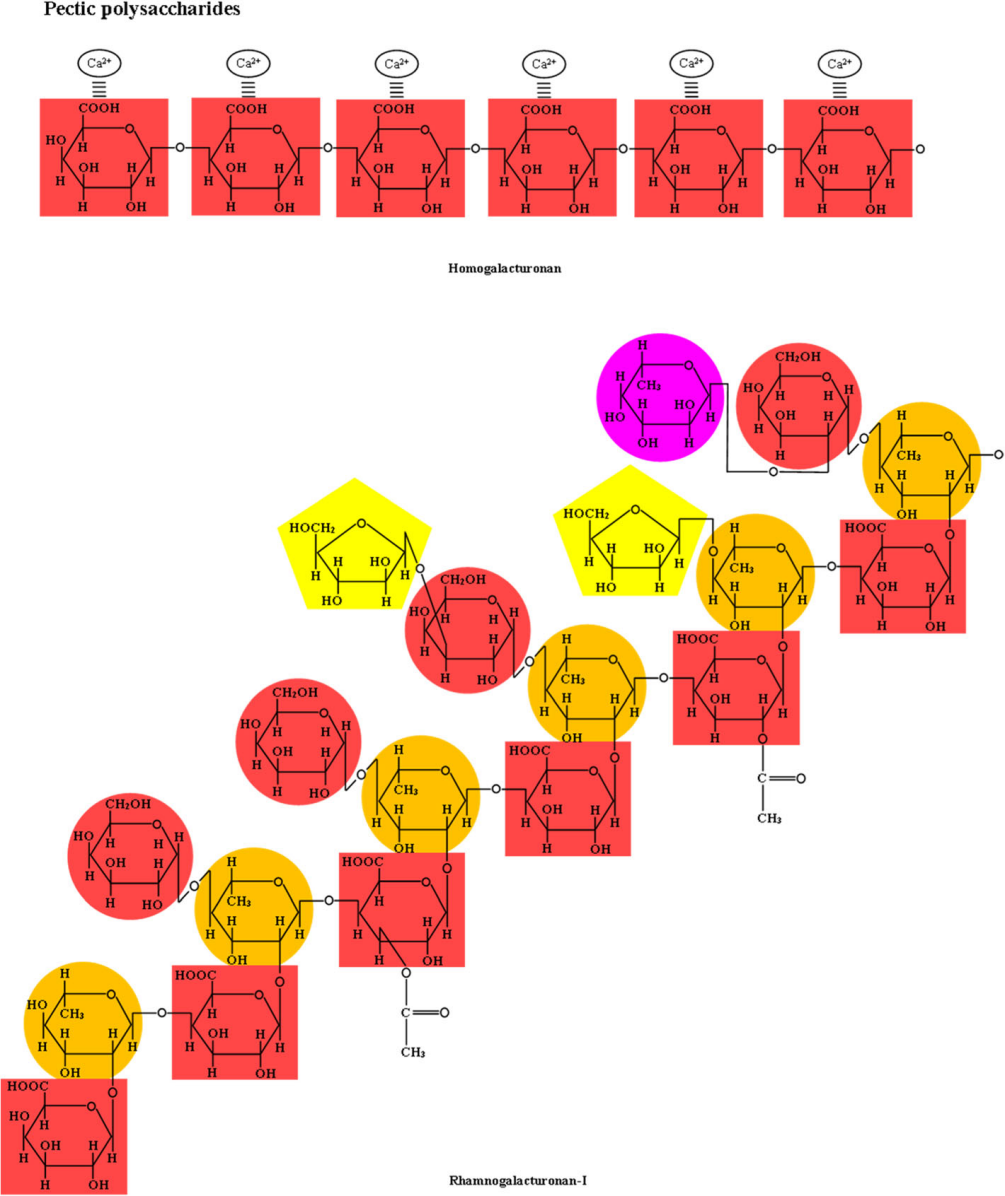
Chemical structure of homogalacturonan and rhamnogalacturonan-I. Adapted from Albersheim et al. [40]

**Fig. 7:**
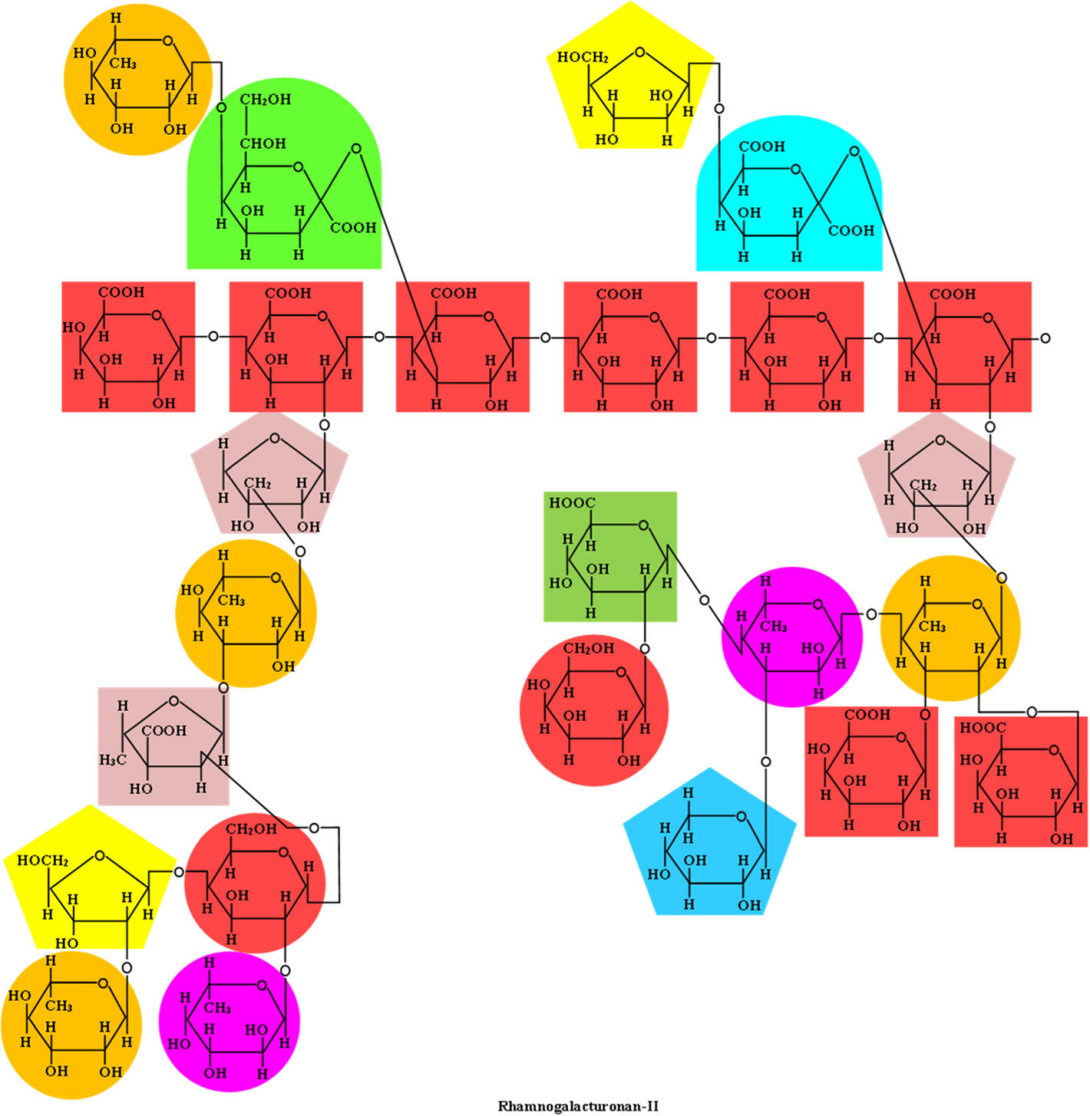
Chemical structure of rhamnogalacturonan-II. Adapted from Albersheim et al. [40]

In addition to cellulose and xyloglucans, primary cell walls also contain pectic polysaccharides that include homogalacturonan and rhamnogalacturonan types I and II [40]. Pectin is a polymer of α-(1,4) linked *d*-galacturonic acid units (homogalacturonan) with uronic acids that may form complexes with Ca and Mg and side-chains that may contain the sugars rhamnose, galactose, arabinose, and xylose (Fig. 6) [42, 46]. The degree and distribution of methyl-esterification at the C-6 carboxyl group and the acetylation at the O-2 and/or O-3 vary among sources [42, 58]. Esterified pectins are located in the cell wall surrounding the cellulose-NCP matrix, while non-esterified homogalacturonan are located predominantly in the middle lamella and cell corner regions [40]. Homogalacturonans can account for 60% of total pectin or greater in plant cell walls and is abundant in potatoes [58]. Rhamnogalacturonan type I (RG-1) is a polymer with an alternating α-(1,2)-*ʟ*-rhamnose and α-(1,4)-*d*-galacturonic acid backbone with side-chains containing α-(1,5)-*ʟ*-arabinans, β-*d*-galactans, and arabinogalactans substituted at the C-4 position [42]. In contrast to homogalacturonan, the *d*-galacturonic acid residues of RG-1 cannot be esterified and may only be acetylated on position 3 [14]. Side-chains of fucosyl, glucosyluronic acid, and 4-O-methyl glucosyluronic acid residues are also present in small amounts in RG-1 [40]. The α-(1,5)-*ʟ*-arabinan side-chains may also have (1,3) branch points, and the β-*d*-galactans that are primarily (1,4) linked may also be occasionally (1,3) linked to the main chain with (1,6) branch points [14]. Solubilized RG-1 from primary cell walls treated with α-1,4-endo-polygalacturonase can account for 5% to 10% of the cell walls of dicotyledons and about 1% of monocotyledons [40]. Rhamnogalacturonan type II (RG-2) has a backbone of α-(1,4)-*d*-galacturonic acid units with aldehydro- and keto-sugar oligosaccharide substitutions at C-2 and C-3 (Fig. 7) [42]. The highly branched RG-2 has approximately 30 glycosyl residues with 11 different monosaccharides, excluding glucose and mannose, making its structure relatively more complex than that of other plant polysaccharides and therefore resistant to microbial fermentation [40]. In addition, uncommon sugars that are associated with RG-2 include 3-deoxy-*d*-manno-oct-2-ulosonic acid, apiose, 2-keto-3-deoxy-*d*-lyxo-heptulosaric acid, and aceric acid [14]. Self-association occurs via a boron diester bond between molecules of RG-2 allowing the formation of dimers [40, 58]. Both RG-1 and RG-2 are covalently linked to the backbone of homoogalacturonan, and it has been suggested that xyloglucans also form covalent cross-linkages with homogalacturonan [58].

**Fig. 8:**
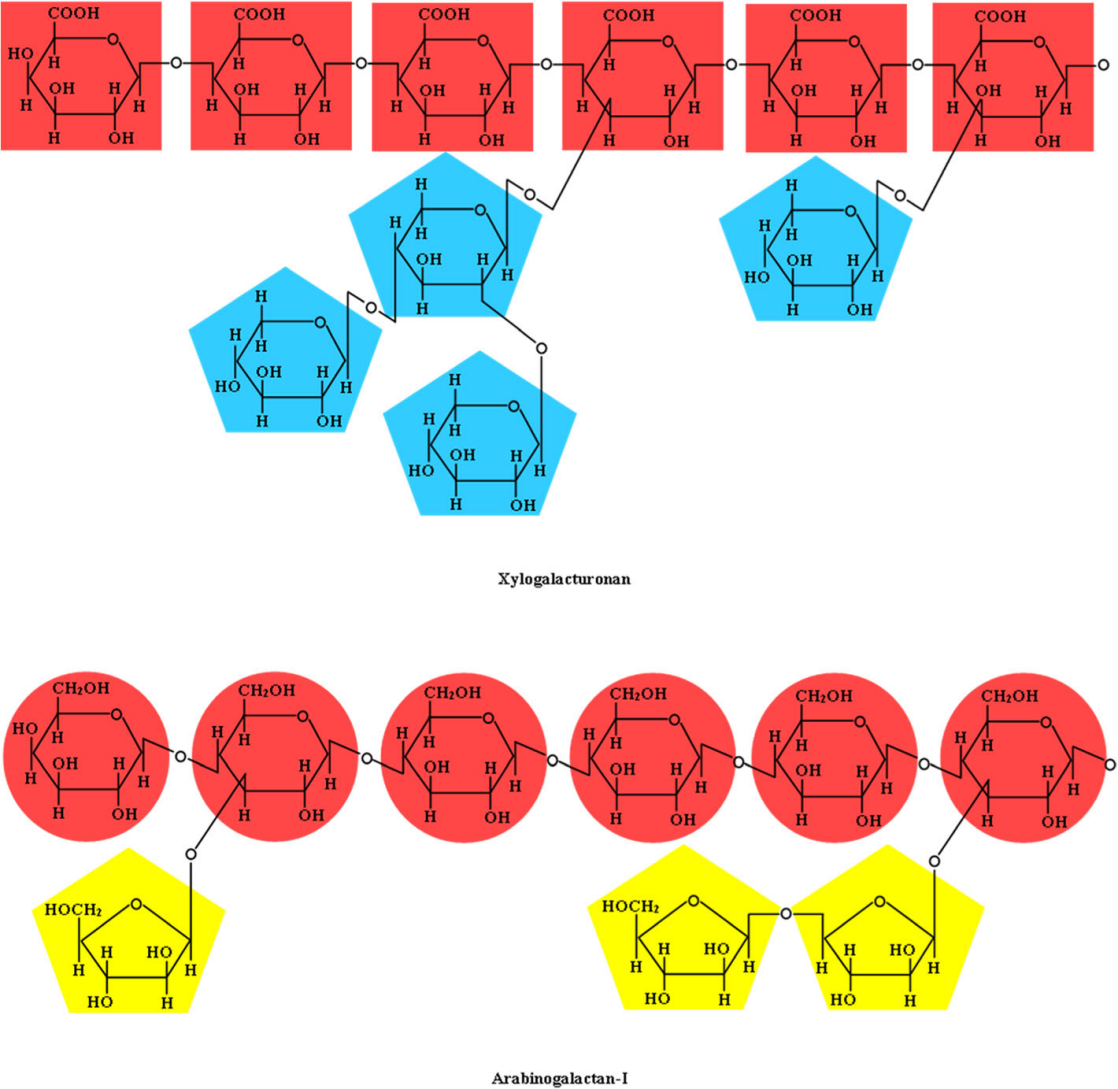
Chemical structure of xylogalacturonan and arabinogalactan-I

**Fig. 9:**
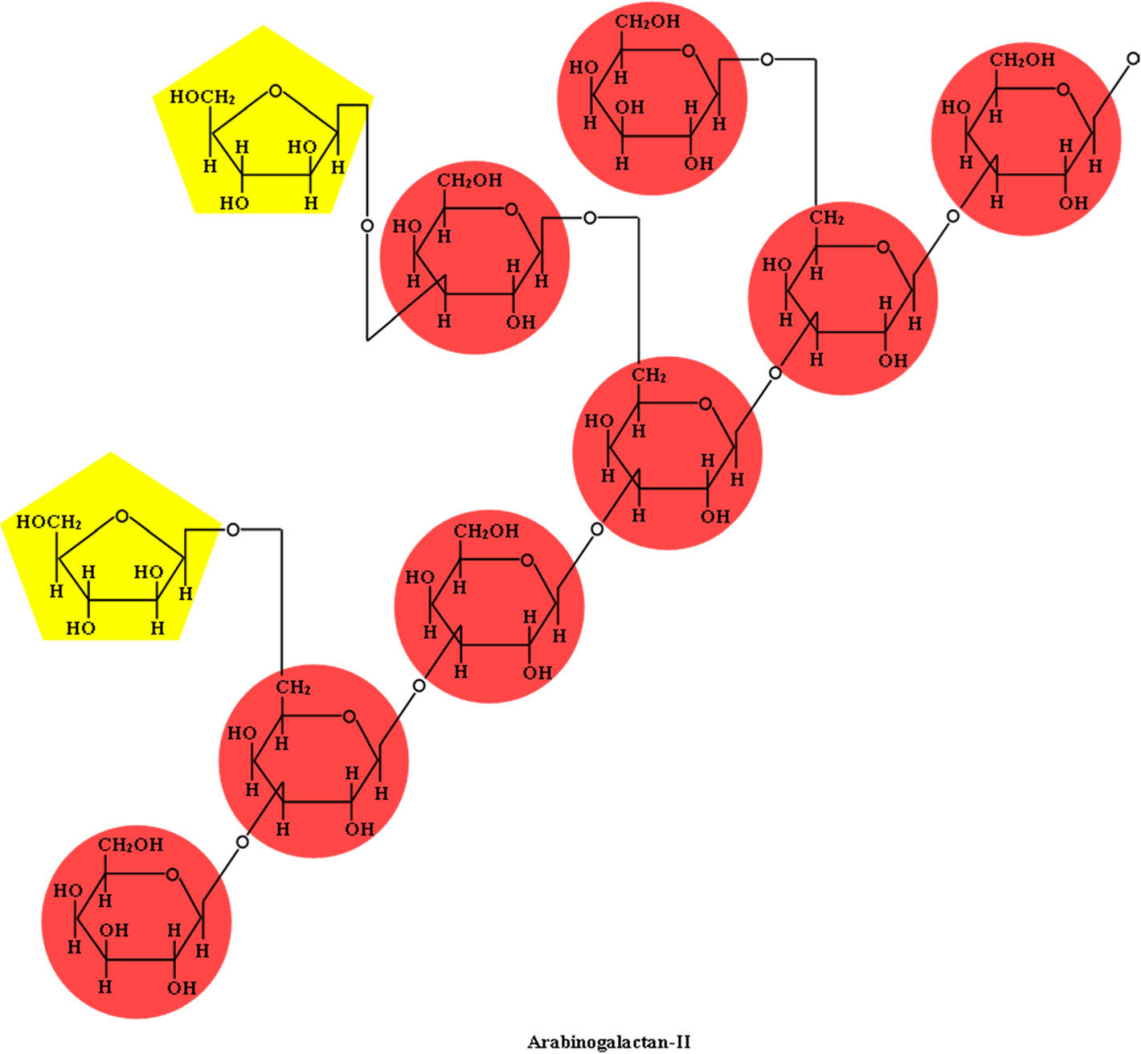
Chemical structure of arabinogalactan-II

Pectic polysaccharides also include xylogalacturonan and arabinogalactans types I and II [42]. Reproductive tissue contains xylogalacturonan, which has a homogalacturonan backbone with one or more β-(1,4)-*d*-xylose residue substitutions at the C-3 position and the first residue is frequently branched at the C-2 by another xylose residue (Fig. 8) [42, 58]. Arabinogalactan types I and II both have linear β-(1,4)-*d*-galactosyl backbones, which may have a short side chain containing α-(1,5)-*ʟ*-arabinoxyl residues (i.e., type I) or have highly branched side-chains containing β-(1,6)-*d*-galactosyl residues (Fig. 9) [42].

Oilseeds are rich sources of protein, but soybeans, along with other legumes, also contain significant quantities of galacto-oligosaccharides, namely raffinose, stachyose, and verbascose. Galacto-oligosaccharides, or α-galactosides, accumulate in storage organs of plants and are only present in the leaves at low concentrations [59]. Among the most common legumes, soybeans have the greatest concentrations of these oligosaccharides, which can make up 5% to 7% of DM (Tables 3 and 4) [21, 60]. Cottonseed products have elevated concentrations of raffinose, whereas soybean meal has the greatest concentrations of stachyose. High concentrations of α-galactosides interfere with digestion of other nutrients and stimulate anaerobic fermentation in the hindgut of humans and pigs that causes flatulence and decreases NE intake [59]. However, fermentation due to the presence of α-galactosides also may have a beneficial effect on ileal lactobacilli and bifidobacteria in the colon and reduce the concentration of colonic enterobacteria [60].

**Table 3:** Carbohydrates and lignin in oilseed meals and expellers (g/kg DM)^a^

Items	Meal					Expellers		
	Soybean	Rapeseed	Canola	Cottonseed	Sunflower	Rapeseed	Cottonseed	Sunflower
Starch	27	18	21	19	23	15	18	10
Cellulose	59	52	112	90	124	59	92	123
Lignin	18	133	83	92	130	90	83	133
Sucrose	70	58	77	16	–	68	10	36
Raffinose	10	4	7	35	–	3	39	14
Stachyose	47	12	26	13	–	13	14	3
Verbascose	3	0	–	2	–	0	1	0
Pectin	68	97	–	–	56	–	–	–
Dietary fiber^b^								
Total dietary fiber	191	–	297	–	–	–	–	–
Insoluble dietary fiber	174	–	286	–	–	–	–	–
Soluble dietary fiber	16	–	11	–	–	–	–	–

^a^Adapted from Bach Knudsen [48], Malathi and Devegowda [71], Bach Knudsen [30], NRC [17], and Bach Knudsen [42], Navarro et al. [66]

^b^Values for dietary fiber were adapted from Jaworski and Stein [69] Navarro et al. [66], Navarro et al. [67]

**Table 4:** Carbohydrates and lignin in pulse crops (g/kg DM)^a, b^

Items	Peas	Lupins	Faba bean	Lentils
Total MBG	ND	–	–	ND
Total AX	11	–	–	10
Starch	432	14	375	598
Resistant starch	22	–	32	74
Cellulose	53	131	81	54
Lignin	12	12	20	–
Sucrose	30	24	27	29
Raffinose	5	10	4	5
Stachyose	23	53	16	37
Verbascose	22	14	34	–
Pectin	8	–	11	–

^a^Adapted from Frias et al. [72], Bach Knudsen [48], Bailoni et al. [64], Bach Knudsen [30], Dodevska et al. [73], and Bach Knudsen [42]

^b^*MBG* mixed linked β-glucan, *AX* arabinoxylan, *ND* not detected

### Pulse crops

Pulse crops, which include beans, lentils, lupins, and peas, are legumes that are rich sources of protein and other nutrients [61]. Peas, faba beans, and lupins are the major pulse crops used as sources of both protein and energy in diets fed to pigs [62]. Relatively high amounts of starch in peas, faba beans, and lentils make them possible alternative sources of energy (Table 4). Similar to oilseed crops, the cell walls of pulse crops contain a variety of polysaccharides that play a role in protection including high concentrations of cellulose, lignin, xyloglucans, and pectin [42]. Pulse crops contain considerable quantities of galacto-oligosaccharides (raffinose, stachyose, and verbascose). Lupins do not contain starch, but have greater concentrations of cellulose, raffinose, and stachyose than the other pulse crops, which may stimulate more microbial fermentation in the hindgut. Verbascose is present in pulse crops in amounts greater than in oilseeds.

## Conclusions

There are limited robust and practical methods to quantify fractions of fiber that are of importance for assessing the energy value of fiber. The chemical properties of fiber fractions have nutritional consequences and alters the physiological conditions in the gastrointestinal tract of pigs. Determination of how the measurable chemical characteristics of the fiber components of feed ingredients influence energy and nutrient digestibility will enable more accurate diet formulation. Therefore, the swine industry will benefit from an improvement in the utilization of energy from less expensive fibrous feed ingredients, and this will result in a more sustainable pork production system due to the reduction in reliance on energy from more costly cereal grains.

## Abbreviations

AX: Arabinoxylans
DP: Degree of polymerization
IDF: Insoluble dietary fiber
MBG: Mixed linked beta-glucans
NCP: Non-cellulosic polysaccharides
NSP: Non-starch polysaccharides
RG-1: Rhamnogalacturonan type I
RG-2: Rhamnogalacturonan type II
RS: Resistant starch
SCFA: Short chained fatty acids
SDF: Soluble dietary fiber

## References

[1] Bach Knudsen KE, Lærke HN, Jørgensen H, Chiba LI (2013). Carbohydrates and carbohydrate utilization in swine. Sustainable swine nutrition.

[2] Slavin JL, Stipanuk MH, Caudill MA (2013). Structure, nomenclature, and properties of carbohydrates. Biochemical, physiological, and molecular aspects of human nutrition.

[3] Ferrier DR (2014). Biochemistry.

[4] Aimutis WR, Polzin K, Paeschke TM, Aimutis WR (2011). The gastrointestinal tract and its microflora. Nondigestible carbohydrates and digestive health.

[5] Paeschke TM, Aimutis WR, Paeschke TM, Aimutis WR (2011). Introduction to fiber and nondigestible carbohydrates: definition, health aspects, and perspectives. Nondigestible carbohydrates and digestive health.

[6] Mateos-Aparicio I, Redondo-Cuenca A, Villanueva MJ, Betancur-Ancona D, Chel-Guerrero L, Segura-Campos MR (2013). Dietary fiber from the food industry by-products. Dietary fiber: sources, properties and their relationship to health.

[7] Urriola PE, Shurson GC, Stein HH (2010). Digestibility of dietary fiber in distillers coproducts fed to growing pigs. J Anim Sci.

[8] Fuller MF (2004). The encyclopedia of farm animal nutrition.

[9] Ouwehand AC, Derrien M, de Vos W, Tiihonen K, Rautonen N (2005). Prebiotics and other microbial substrates for gut functionality. Curr Opin Biotechnol.

[10] Qaisrani SN, Moquet PCA, van Krimpen MM, Kwakkel RP, Verstegen MWA, Hendriks WH (2014). Protein source and dietary structure influence growth performance, gut morphology, and hindgut fermentation characteristics in broilers. Poult Sci.

[11] BeMiller JN, Embuscado ME (2014). Essentials of carbohydrate chemistry. Functionalizing carbohydrates for food applications: texturizing and bioactive/flavor delivery systems.

[12] Vaclavik VA, Christian EW (2014). Essentials of food science.

[13] BeMiller JN (2007). Carbohydrate chemistry for food scientists.

[14] Sinnott M (2013). Carbohydrate chemistry and biochemistry: structure and mechanism.

[15] Englyst KN, Liu S, Englyst HN (2007). Nutritional characterization and measurement of dietary carbohydrates. Eur J Clin Nutr.

[16] Nguema-Ona E, Vicre-Gibouin M, Gotte M, Plancot B, Lerouge P, Bardor M (2014). Cell wall O-glycoproteins and N-glycoproteins: aspects of biosynthesis and function. Front Plant Sci.

[17] NRC (2012). Nutrient requirements of swine.

[18] Leturque A, Brot-Laroche E, Stipanuk MH, Caudill MA (2013). Digestion and absorption of carbohydrates. Biochemical, physiological, and molecular aspects of human nutrition.

[19] Dumville JC, Fry SC (2003). Gentiobiose: a novel oligosaccharin in ripening tomato fruit. Planta..

[20] Roberfroid M, Slavin J (2000). Nondigestible oligosaccharides. Crit Rev Food Sci Nutr.

[21] Liener IE, Drackley JK (2000). Non-nutritive factors and bioactive compounds in soy. Soy in animal nutrition.

[22] Marin-Manzano MC, Abecia L, Hernandez-Hernandez O, Sanz ML, Montilla A, Olano A (2013). Galacto-oligosaccharides derived from lactulose exert a selective stimulation on the growth of *bifidobacterium animalis* in the large intestine of growing rats. J Agric Food Chem.

[23] Cromwell GL, Chiba LI (2013). Feed additives in swine diets. Sustainable swine nutrition.

[24] Han YW (1990). Microbial levan. Adv App Microbiol.

[25] Chawla R, Patil GR (2010). Soluble dietary fiber. Compr Rev Food Sci Food Saf.

[26] Vijn I, Smeekens S (1999). Fructan: more than a reserve carbohydrate. Plant Physiol.

[27] Van den Ende W (2013). Multifunctional fructans and raffinose family oligosaccharides. Front Plant Sci.

[28] Swanson KS, Grieshop CM, Flickinger EA, Merchen NR, Fahey GC (2002). Effects of supplemental fructooligosaccharides and mannanoligosaccharides on colonic microbial populations, immune function and fecal odor components in the canine. J Nutr.

[29] Che TM, Johnson RW, Kelley KW, Dawson KA, Moran CA, Pettigrew JE (2012). Effects of mannan oligosaccharide on cytokine secretions by porcine alveolar macrophages and serum cytokine concentrations in nursery pigs. J Anim Sci.

[30] Bach Knudsen KE (2011). Triennial growth symposium: effects of polymeric carbohydrates on growth and development of pigs. J Anim Sci.

[31] Kiem NL, Levin RJ, Havel PJ, Ross AC, Caballero B, Cousins RJ, Tucker KL, Ziegler TR (2014). Carbohydrates. Modern nutrition in health and disease.

[32] Dar YL, Embuscado ME (2014). Starches as food texturizing systems. Functionalizing carbohydrates for food applications: texturizing and bioactive/flavor delivery systems.

[33] Serna-Saldivar SO (2010). Cereal grains: properties, processing, and nutritional attributes.

[34] Hill PG, Burtis CA, Ashwood ER, Bruns DE (2006). Gastric, pancreatic, and intestinal function. Clinical chemistry and molecular diagnostics.

[35] Sacks DB, Burtis CA, Ashwood ER, Bruns DE (2006). Carbohydrates. Clinical chemistry and molecular diagnostics.

[36] Brown IL (2004). Applications and uses of resistant starch. J AOAC Int.

[37] Cervantes-Pahm SK, Liu Y, Stein HH (2014). Comparative digestibility of energy and nutrients and fermentability of dietary fiber in eight cereal grains fed to pigs. J Sci Food Agric.

[38] DeVries JW (2004). Dietary fiber: the influence of definition on analysis and regulation. J AOAC Int.

[39] McGrane MM, Stipanuk MH, Caudill MA (2013). Carbohydrate metabolism: synthesis and oxidation. Biochemical, physiological, and molecular aspects of human nutrition.

[40] Albersheim P, Darvill A, Roberts K, Sederoff R, Staehelin A (2011). Plant cell walls.

[41] Pluske JR, Kim JC, McDonald DE, Pethick DW, Hampson DJ, Varley MA, Wiseman J (2001). Non-starch polysaccharides in the diets of young weaned piglets. The weaner pig: nutrition and management.

[42] Bach Knudsen KE (2014). Fiber and nonstarch polysaccharide content and variation in common crops used in broiler diets. Poult Sci.

[43] Brett C, Waldron K (1990). Physiology and biochemistry of plant cell walls.

[44] Bhat MK, Hazlewood GP, Bedford MR, Partridge GG (2001). Enzymology and other characteristics of cellulases and xylanases. Enzymes in farm animal nutrition.

[45] Paloheimo M, Piironen J, Vehmaanpera J, Bedford M, Partridge G (2010). Xylanases and cellulases as feed additives. Enzymes in farm animal nutrition.

[46] Cummings JH, Stephen AM (2007). Carbohydrate terminology and classification. Eur J Clin Nutr.

[47] Boudet AM, Rose JKC (2003). Towards an understanding of the supramolecular organization of the lignified wall. The plant cell wall.

[48] Bach Knudsen KE (1997). Carbohydrate and lignin contents of plant materials used in animal feeding. J Anim Feed Sci Technol.

[49] Slavin JL, Stipanuk MH, Caudill MA (2013). Dietary fiber. Biochemical, physiological, and molecular aspects of human nutrition.

[50] Izydorczyk MS, Biliaderis CG, Biliaderis CG, Izydorczyk MS (2007). Arabinoxylans: technologically and nutritionally functional plant polysaccharides. Functional food carbohydrates.

[51] Pritchard JR, Lawrence GJ, Larroque O, Li Z, Laidlaw HKC, Morell MK (2011). A survey of β-glucan and arabinoxylan content in wheat. J Sci Food Agric.

[52] Izydorczyk MS, Biliaderis CG (1995). Cereal arabinoxylans: advances in structure and physicochemical properties. Carbohydr Polym.

[53] Izydorczyk MS, Dexter JE (2008). Barley β-glucans and arabinoxylans: molecular structure, physicochemical properties, and uses in food products – a review. Food Res Int.

[54] Sinha AK, Kumar V, Makkar HPS, Boeck GD, Becker K (2011). Non-starch polysaccharides and their role in fish nutrition – a review. Food Chem.

[55] Lazaridou A, Biliaderis CG, Izydorczyk MS, Biliaderis CG, Izydorczyk MS (2007). Cereal β-glucans: structures, physical properties, and physiological functions. Functional food carbohydrates.

[56] Smith BG, Melton LD, Wrolstad RE (2012). Plant cell wall polysaccharides. Food carbohydrate chemistry.

[57] O’Neill MA, York WS, Rose JKC (2003). The composition and structure of plant primary cell walls. The plant cell wall.

[58] Caffall KH, Mohnen D (2009). The structure, function, and biosynthesis of plant cell wall pectic polysaccharides. Carbohydr Res.

[59] Martinez-Villaluenga C, Frias J, Vidal-Valverde C (2008). Alpha-galactosides: antinutritional factors or functional ingredients?. Crit Rev Food Sci Nutr.

[60] Middelbos IS, Fahey GC, Johnson LA, White PJ, Galloway R (2008). Soybean carbohydrates. Soybeans: chemistry, production processing, and utilization.

[61] Maiti R, Satya P, Rajkumar D, Ramaswamy A (2012). Crop plant anatomy.

[62] Aumiller T, Mosenthin R, Weiss E (2015). Potential of cereal grains and grain legumes in modulating pigs’ intestinal microbiota – a review. Livestock Sci.

[63] McCleary BV, Glennie-Holmes M (1985). Enzymic quantification of (1→3) (1→4)-β-d-glucan in barley and malt. J Inst Brew.

[64] Bailoni L, Bonsembiante M, Schiavon S, Pagnin G, Tagliapietra F (2003). Estimation of the content of pectins in feeds: Fractional extraction and quantitative determination. Vet Res Comm.

[65] Jaworski NW, Lærke HN, Bach Knudsen KE, Stein HH (2015). Carbohydrate composition and in vitro digestibility of dry matter and nonstarch polysaccharides in corn, sorghum, and wheat and coproducts from these grains. J Anim Sci.

[66] Navarro DMDL, Bruininx EMAM, de Jong L, Stein HH (2018). Analysis for low-molecular-weight carbohydrates is needed to account for all energy-contributing nutrients in some feed ingredients, but physical characteristics do not predict in vitro digestibility of dry matter. J Anim Sci.

[67] Navarro DMDL, Bruininx EMAM, de Jong L, Stein HH (2018). The contribution of digestible and metabolizable energy from high-fiber dietary ingredients is not affected by inclusion rate in mixed diets fed to growing pigs. J Anim Sci.

[68] Curry SM, Navarro DMDL, Almeida FN, Almeida JAS, Stein HH (2014). Amino acid digestibility in low-fat distillers dried grains with solubles fed to growing pigs. J Anim Sci Biotechnol.

[69] Jaworski NW, Stein HH (2017). Disappearance of nutrients and energy in the stomach and small intestine, cecum, and colon of pigs fed corn-soybean meal diets containing distillers dried grains with solubles, wheat middlings, or soybean hulls. J Anim Sci.

[70] McCleary BV, DeVries JW, Rader JI, Cohen G, Prosky L, Mugford DC (2012). Determination of insoluble, soluble, and total dietary fiber (CODEX definition) by enzymatic-gravimetric method and liquid chromatography: collaborative study. J AOAC Int.

[71] Malathi V, Devegowda G (2001). In vitro evaluation of nonstarch polysaccharide digestibility of feed ingredients by enzymes. Poult Sci.

[72] Frias J, Vidal-Valverde C, Kozlowska H, Tabera J, Honke J, Hedley CL (1996). Natural fermentation of lentils. Influence of time, flour concentration, and temperature on the kinetics of monosaccharides, disaccharide, and α-galactosides. J Agric Food Chem.

[73] Dodevska MS, Djordjevic BI, Sobajic SS, Miletic ID, Djordjevic PB, Dimitrijevic-Sreckovic VS (2013). Characterisation of dietary fibre components in cereals and legumes used in Serbian diet. Food Chem.

